# Stimulating the Auto-Protective Processes of the Body

**Published:** 1910-04

**Authors:** 


### Stimulating the Auto-Protective Processes of the Body.

The trend of medical thought is unquestionably towards organic medication. In the last edition of Osler's "Modern Medicine" we find the following: "However produced, the ultimate processes of disease represent chemical changes in the fluids and tissues of the body, and in this direction, too, the advances of the past half century have had a profound influence on chemical medication."

Our knowledge of normal metabolism has progressed with startling rapidity, and warrants the belief that before long we shall have a safe platform from which to investigate "to a finish" such serious perversions as are present in gout, diabetes, etc. Already the studies upon internal secretions have not only given us a clear conception of the functions of certain glands, but have also enabled us to treat successfully such otherwise incurable maladies as myxedema. In the immediate future, it is along chemical lines that we may look for the greatest advance, and of this there is no more certain indication than the simultaneous appearance quite recently in England and the United States of journals devoted to biochemistry.

Sajous, an eminent writer on therapeutics, who has made the internal secretions a special study, says, "We should look to the auto-protective resources of the body and the laws through which drugs influence them for a scientific therapeusis."

In a volume of *Progressive Medicine,* June, 1909, Alfred Stengel, M. D., dealing with the etiology, pathology, and treatment of exophthalmic goitre, pays this tribute to the place the internal secretions are winning in our newer conception of the treatment of disease: "During the last year considerable attention has been devoted to the relation of the activity of the other glands of the body with an internal secretion to the function of the thyroid gland. Hoffman believes that the thyroid and the suprarenals are antagonists, and that the former has much to do with the functioning of the splanchnic nerve. The hypophysis can act vicariously for the thyroid to a certain extent, while the functioning of the ovary rises and falls with that of the thyroid.

"Extract of testicle acts like suprarenal extract in increasing muscular energy. When a gland with internal secretion is doing extra work, its antagonist also has to increase its functioning to keep up with it, or the balance is lost. Excessive functioning of the one gland is accompanied by deficient functioning in its antagonist. The practical results of this study are that the affections of various glands should be treated with organotherapy from the antagonist glands. He has found the serum of (thyroidectomized) sheep of value in the treatment of osteomalacia, mongolism and abortive forms of myxedema. He thinks that ovarian and testicular extract would probably prove still more effective in these latter cases."

"Krauss and Friedenthal have also done considerable research work to confirm the physiological antagonistic action of the internal secretions."

The brilliant achievements of the serums and bacterial vaccines in the treatment of diseases of microbic origin have stimulated to a remarkable degree further study and investigation into the action of the agents (termed by Oswald, Katalystators) able to influence temporarily metabolic changes within the body.

The proper study of metabolism considers three objects:

1. Investigation of the "exchange of materials"—(Stoffwechsel)—and the extent and character of the incessant transformations which accompany and determine the changes occurring within the living tissues.

2. It aims to ascertain the "conversion of chemical tension into living energy."

3. How the normal process is affected by the disturbance of the proper balance which should obtain in the secretions.

It has been conclusively demonstrated that such animal derivatives as the thyreoid, suprarenal, ovarian, orchic and lymphatic substance, pepsin pancreatin, etc., will, when orally administered, supply definite compensatory elements in the human economy. The antagonistic action of the internal secretions has been established by the researches by Krauss, Friedenthal and others. It is conceded by practically all of the authorities that if we introduce certain of the animal substances with an internal secretion, we will produce an excess of this secretion within the body, and, therefore, stimulate certain antagonistic secretions which may not have been functioning properly. It requires very simple reasoning to figure that if by such stimulation we can restore that perfect harmony which in normal health obtains any morbid condition, must, of necessity, be benefited.

Dr. Arthur Latham, of London, England, has made an exhaustive study of the immunizing effect of passing tuberculin and other bacterial emulsions through the lining walls of the intestinal tract. He has clearly demonstrated that such action is possible, and that positive antibodies may be developed by this method. Our own researches have demonstrated that certain of the animal derivatives combined with drug synergists decidedly increase the leucocytes, and have a definite compensatory and tonic action upon the cell tissues.

Extensive experiments have been conducted with what are assumed to be the active elements of a number of the animal derivatives, but the results obtained from their clinical employment has not been the same as the action of the substance itself; as examples of this we may cite the independent use of such agents as nuclein, lecithin, cholesterin, thyroidin, etc. Having established the definite tonic action of certain animal derivatives upon metabolic processes, we have incorporated in what we consider the newest addition to animal therapy, substances which our research has demonstrated possess the greatest remedial activity. With the animal substances, we have included drug synergists which are important constituents of the normal cells or have a selective action upon the nervous system or the eliminative process.

---

Osteosarcoma about a joint may closely simulate a rapidly-growing exostosis of arthritis deformans.—*American Journal of Surgery.*

